# RNA *N*^6^-methyladenosine modification in cancers: current status and perspectives

**DOI:** 10.1038/s41422-018-0034-6

**Published:** 2018-04-23

**Authors:** Xiaolan Deng, Rui Su, Hengyou Weng, Huilin Huang, Zejuan Li, Jianjun Chen

**Affiliations:** 1 Department of Systems Biology & the Gehr Family Center for Leukemia Research, The Beckman Research Institute of City of Hope, Monrovia, CA 91016 USA; 2 School of Pharmacy, China Medical University, Shenyang, 110122 China; 3 Department of Cancer Biology, University of Cincinnati College of Medicine, Cincinnati, OH 45219 USA; 4 Department of Human Genetics, University of Chicago, Chicago, IL 60637 USA

## Abstract

*N*^6^-methyladenosine (m^6^A), the most abundant internal modification in eukaryotic messenger RNAs (mRNAs), has been shown to play critical roles in various normal bioprocesses such as tissue development, stem cell self-renewal and differentiation, heat shock or DNA damage response, and maternal-to-zygotic transition. The m^6^A modification is deposited by the m^6^A methyltransferase complex (MTC; i.e., writer) composed of METTL3, METTL14 and WTAP, and probably also VIRMA and RBM15, and can be removed by m^6^A demethylases (i.e., erasers) such as FTO and ALKBH5. The fates of m^6^A-modified mRNAs rely on the functions of distinct proteins that recognize them (i.e., readers), which may affect the stability, splicing, and/or translation of target mRNAs. Given the functional importance of the m^6^A modification machinery in normal bioprocesses, it is not surprising that evidence is emerging that dysregulation of m^6^A modification and the associated proteins also contributes to the initiation, progression, and drug response of cancers. In this review, we focus on recent advances in the study of biological functions and the underlying molecular mechanisms of dysregulated m^6^A modification and the associated machinery in the pathogenesis and drug response of various types of cancers. In addition, we also discuss possible therapeutic interventions against the dysregulated m^6^A machinery to treat cancers.

## Introduction

It is well known that gene expression and cell growth/division are under sophisticated controls through genetic and epigenetic regulations. Abnormal genetic changes (e.g., gene mutation, deletion, amplification, or chromosomal translocation) and/or epigenetic abnormalities (e.g., DNA or histone modification changes) may lead to the development of cancers. In recent years, another layer of gene regulation at the RNA level, i.e., RNA epitranscriptomics,^1^ has gained increased attention and interest in the research community. Since 1960s, over 100 types of chemical modifications have been identified in protein-coding and non-coding RNAs.^2–4^ Of them, *N*^6^-methyladenosine (m^6^A) is the most abundant internal modification on eukaryotic mRNAs.^5,6^ The identification of the fat mass and obesity-associated protein (FTO) as a genuine demethylase of m^6^A modification^7^ indicated that m^6^A is a reversible and dynamic RNA modification, analogous to the well-studied reversible DNA and histone modifications.^8^ Subsequent high-throughput m^6^A sequencing studies revealed that m^6^A modifications may affect thousands of mRNAs and non-coding RNAs in each given type of cell, with a special enrichment in the 3′ untranslated regions (UTRs) near the stop codons of mRNAs.^9,10^

**Fig. 1:**
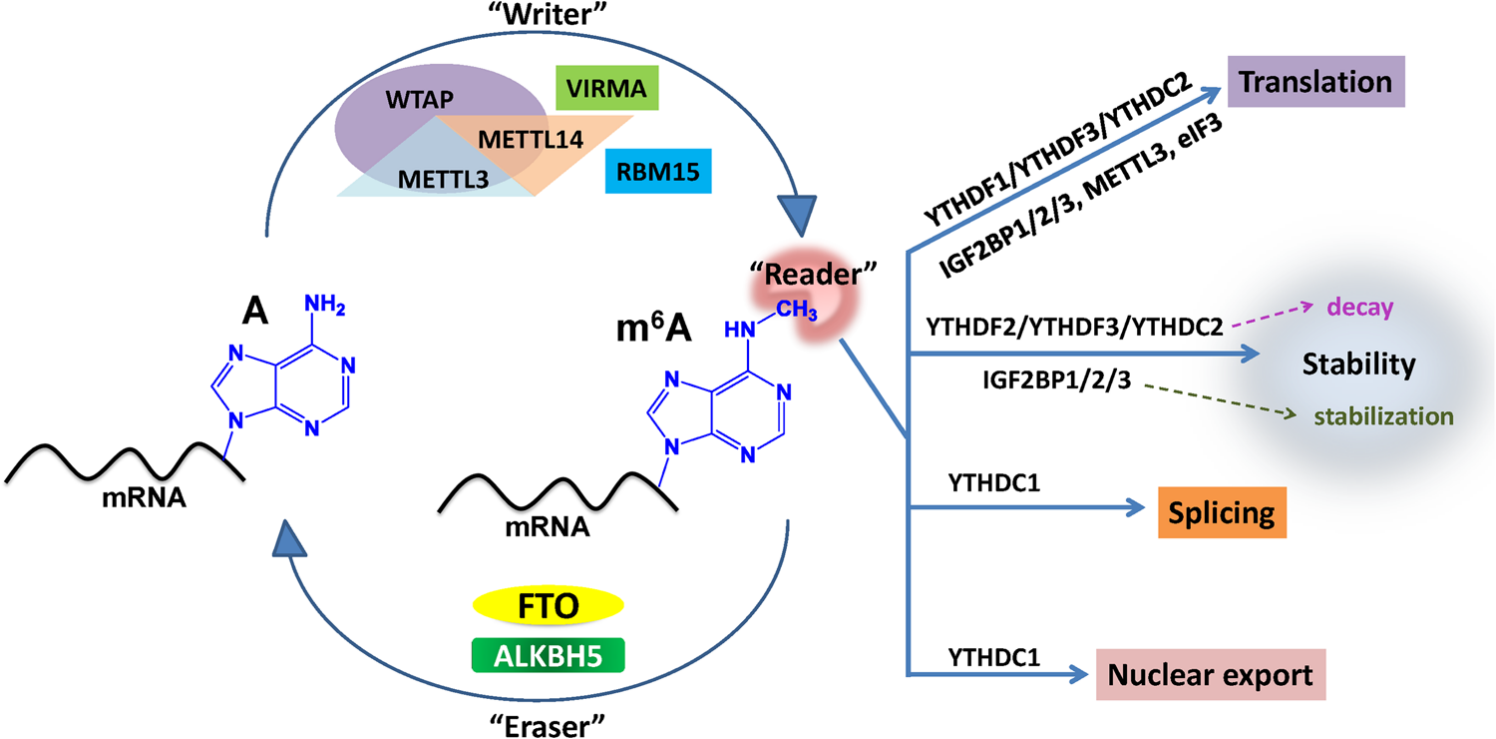
Summary of m^6^A modification machinery. The m^6^A methyltransferase complex composed of METTL13, METTL14 and WTAP, probably also of VIRMA and RBM15, serves as m^6^A “writer”, demethylases (e.g., FTO and ALKBH5) serve as m^6^A “erasers”, and a set of m^6^A-binding proteins (e.g., YTHDF1/2/3, YTHDC1/2, IGF2BP1/2/3, METTL3 and eIF3) serve as m^6^A “readers” that determine the fate of target m^6^A-modified mRNA transcripts

Methyltransferase-like 3 and 14 (METTL3 and METTL14) and their cofactors, Wilms tumor 1-associated protein (WTAP), VIRMA (KIAA1429), and RBM15, compose the m^6^A methyltransferase complex (MTC) that catalyzes m^6^A modification as the m^6^A writer.^11–16^ A set of m^6^A demethylases, such as FTO and ALKBH5, can remove m^6^A modification from RNA as m^6^A erasers and thus keep m^6^A modification in a dynamic balance.^6,7,17^ Members of the YT521-B homology (YTH) domain family of proteins, including YTHDF1, YTHDF2, YTHDF3, YTHDC1, and YTHDC2, have been identified as direct m^6^A readers.^18–23^ While YTHDF2, YTHDF3, and YTHDC2 may promote decay of target mRNAs, YTHDF1, YTHDF3, and YTHDC2 can promote translation of target mRNAs, and YTHDC1 likely impacts splicing and nuclear export of target mRNAs.^18–24^ Notably, in contrast to the decay-promoting functions of YTHDF2, YTHDF3, and YTHDC2, a recently identified new family of m^6^A readers, including IGF2BP1, IGF2BP2, and IGF2BP3, promote the stability (and also translation) of most of their target mRNAs (e.g., *MYC*).^25^ Eukaryotic initiation factor 3 (eIF3) could be considered as a reader of 5′ UTR m^6^A.^26^ It was reported that cytoplasmic METTL3 may also serve as a kind of m^6^A reader and promote translation of target mRNAs in certain cell types.^27^ Thus, dependent on the type of reader protein that recognizes the m^6^A modification of a given target mRNA, the stability of the target mRNA can be either decreased or enhanced, and translation, splicing, or nuclear transport of the target mRNA may also be affected. See Fig. 1 for a summary of the currently known m^6^A modification machinery.

During the past a few years, m^6^A modification in mRNAs or non-coding RNAs has been reported to play a critical role in virtually all major normal bioprocesses including self-renewal and differentiation of embryonic stem cells, tissue development (e.g., neurogenesis and hematopoiesis), response to heat shock or DNA damage, control of circadian clock, spermatogenesis, and maternal-to-zygotic transition, as well as primary microRNA processing, and RNA–protein interactions.^9,10,13,17–19,23,26,28–38^ More recently, extensive efforts have been exerted in investigating the biological impacts of dysregulated m^6^A modification and the associated machinery (i.e., m^6^A writer, eraser, and reader proteins) in various cancers.^39^ In this review, we focus on recent advances in the study of biological functions and underlying molecular mechanisms of dysregulated m^6^A modification and the associated regulatory proteins in the pathogenesis of various types of cancers, including leukemia, brain tumor, breast cancer, liver cancer, cervical cancer, and lung cancer. Moreover, we also discuss potential therapeutic strategies targeting dysregulated m^6^A machinery to treat the associated cancers.

## FTO functions as an oncogenic m^6^A demethylase in leukemia and brain tumor

FTO became very famous a decade ago due to the strong association of single nucleotide polymorphisms (SNPs) located in its genomic locus with overweight and obesity in humans identified by large-scale, genome-wide association studies.^40–43^ Although there are some controversial discoveries regarding the link of FTO with overweight and obesity,^44,45^ mouse model studies did suggest a critical role of FTO in regulating fat mass, adipogenesis, and body weight,^46–48^ and there is also a link between the SNP risk genotype and increased *FTO* expression in human fibroblasts and blood cells.^49,50^ In addition, large-scale epidemiology studies demonstrate people with *FTO* SNPs or overweight/obesity have a higher risk of development of cancers such as breast, kidney, prostate, and pancreatic cancers, as well as leukemia, lymphoma and myeloma.^51–54^ However, the definitive role of FTO in cancer remained elusive.

**Fig. 2:**
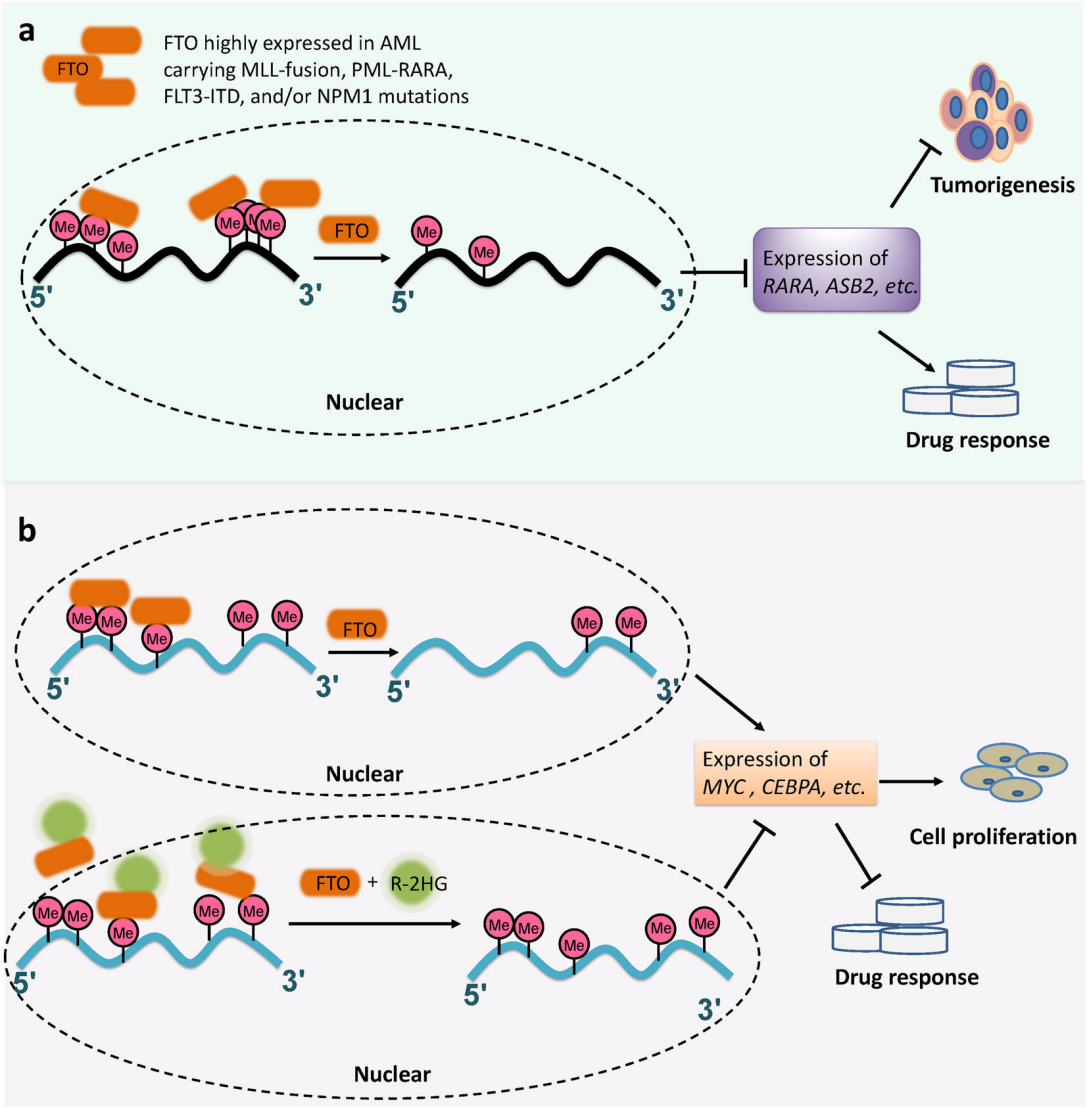
FTO plays a critical oncogenic role in cancer as an m^6^A eraser and its function can be suppressed by R-2HG. **a** FTO functions as an oncogenic m^6^A demethylase in acute myeloid leukemia. **b** R-2HG targets the FTO/m6A/MYC/CEBPA axis and displays anti-tumor effects in leukemia and brain tumor

To investigate the pathological role of FTO in cancer, we analyzed genome-wide gene expression profiling datasets of several large-cohorts of patients with primary acute myeloid leukemia (AML), and found that *FTO* is highly expressed in certain subtypes of AMLs including those carrying t(11q23)/*MLL*-rearrangements, t(15;17)/*PML-RARA*, *FLT3*-ITD, and/or *NPM1* mutations.^55^ We next conducted in vitro and in vivo gain- and loss-of-function studies and demonstrated that forced expression of FTO enhanced human AML cell survival and proliferation, promoted leukemic oncogene (e.g., MLL-AF9) mediated transformation of normal hematopoietic stem/progenitor cells (HSPCs) and leukemogenesis, and inhibited all-trans retinoic acid (ATRA)-induced AML cell differentiation; the opposite was true when *FTO* expression was depleted.^55^ Thus, our data demonstrated that FTO plays an essential oncogenic role in cell transformation and leukemogenesis, as well as in drug response of AML cells. Importantly, we showed that FTO exerts its oncogenic role in AML in an m^6^A-dependent manner as an m^6^A demethylase.^55^ Briefly, FTO post-transcriptionally regulates the expression of its critical target RNAs, such as *ASB2* and *RARA*, two genes that have been implicated in leukemia cell proliferation and drug response.^56–58^ We performed transcriptome-wide m^6^A-seq, luciferase reporter and mutagenesis assays, mRNA stability assays and gene-specific m^6^A-qPCR assays. Data were presented to show that FTO negatively regulates the expression of *ASB2* and *RARA* through reducing the abundance of internal m^6^A modification, especially in the 3′ untranslated regions (3′-UTRs), which in turn leads to decreased stability of the target mRNA transcripts.^55^ Overall, our work provides compelling evidence showing the functional importance of m^6^A modification and FTO in tumorigenesis and drug response (see Fig. 2a).

In brain tumor, Cui et al.^60^ reported that pharmaceutical inhibition of FTO by a chemical inhibitor (MA2, the ethyl ester form of meclofenamic acid (MA), a US Food and Drug Administration (FDA)-approved nonsteroidal anti-inflammatory drug that was shown to be a selective inhibitor of FTO^59^) suppresses tumor progression and substantially prolongs the lifespan of glioblastoma (GBM) stem cell (GSC)-grafted mice. Thus, FTO likely also plays a critical oncogenic role in self-renewal of GSCs and is required for the development of GBM.

## R-2HG targets the FTO/m^6^A/MYC/CEBPA axis and displays anti-tumor effects in leukemia and brain tumor

R-2-hydroxyglutarate (R-2HG), produced at high levels by mutant isocitrate dehydrogenase 1/2 (IDH1/2) enzymes, which could be found in 10–20% of AML patients, ~80% of grade II-III gliomas and secondary GBM, and at a lower rate in other cancers, was reported as an oncometabolite.^61–68^ For instance, mutant IDH1 and its product R-2HG induce cytokine-independent growth and block erythropoietin (EPO)-mediated differentiation of TF-1 cells, a unique erythroleukemia cell line that is GM-CSF-dependent.^68^ Surprisingly, through analysis of the effects of R-2HG on cell viability, proliferation, apoptosis and cell cycle in 27 human leukemia cell lines, 15 primary AML samples, and 8 human GBM cell lines, we very recently found that R-2HG actually displays a broad and intrinsic anti-tumor activity in leukemia and glioma, causing decreased cancer cell viability/proliferation and increased cell-cycle arrest and apoptosis in a time- and dose-dependent manner in the vast majority of the tested samples.^69^ Exogenous R-2HG treatment showed no noticeable inhibitory effects on viability/proliferation of IDH-mutant AML cells, indicating these cells can tolerate the potential inhibitory effect of R-2HG. Moreover, we employed “human-in-mouse” xeno-transplantation leukemic models to evaluate the effect of R-2HG on leukemia progression in vivo. We found that both exogenous (in vivo injected) and endogenous (IDH1^R132H^-generated) R-2HG substantially inhibited leukemia progression in recipient mice xeno-transplanted with 2HG-sensitive AML cells (e.g., NOMO-1 or MA9.3ITD^70^), which was associated with reduced splenomegaly and inhibited engraftments in peripheral blood, bone marrow and spleen. However, no significant inhibitory effects were observed in mice xeno-transplanted with 2HG-resistant AML cells (e.g., MA9.3RAS^70^ or NB4 cells).^69^

Mechanistically, we identified FTO as a direct target of R-2HG and a main mediator of R-2HG-induced anti-tumor effects. R-2HG binds directly to FTO protein and inhibits its m^6^A demethylase activity, resulting in a significant increase of global m^6^A abundance in R-2HG-sensitive leukemia cells, and the effects of R-2HG is FTO-dependent. Notably, *MYC* is a direct and functionally essential target of FTO, and R-2HG treatment or *FTO* knockdown increases m^6^A level on *MYC* mRNA (especially at the 5′ UTR and middle exons), leading to mRNA decay and *MYC* down-regulation, and thereby suppression of MYC signaling.^69^ Interestingly, *FTO* transcription is controlled by CEBPA, and *CEBPA* mRNA is a direct target of FTO and is positively regulated by FTO in an m^6^A-dependent manner, so that there is a positive reciprocal regulation between FTO and CEBPA; as a result, R-2HG treatment can indirectly downregulate expression of both *CEBPA* and *FTO* through the FTO/m^6^A/CEBPA/FTO circuit.^69^ S-2HG, the enantiomer of R-2HG, exhibits similar effects to R-2HG by direct targeting FTO, causing increased global m^6^A modification and decreased leukemic cell proliferation/viability. Our data indicate that FTO/MYC homeostasis controls the sensitivity of leukemic cells to 2HG: a high abundance of FTO confers 2HG sensitivity in leukemic cells, whereas hyper-activation of MYC-associated signaling pathways renders leukemic cells resistant to 2HG; pharmaceutical or genetic inhibition of MYC signaling (e.g., by JQ1 or *MYC* shRNAs) can sensitize 2HG-resistant leukemic cells to (exogenous and endogenous) 2HG.^69^

Moreover, R-2HG also exhibits a synergistic effect with a series of first-line chemotherapy drugs such as ATRA, Azacitidine (AZA), Decitabine, and Daunorubicin. The inhibitory effect of R-2HG is also found in human brain tumor cells. Collectively, our results uncover a new mechanism involving an R-2HG**⊣**FTO**⊣**m^6^A**⊣**MYC**/**CEBPA axis that impacts cancer pathogenesis and drug response through post-transcriptional RNA methylation regulation (see Fig. 2b).^69^

Based on our work and those published by others, we presumed that endogenous R-2HG in IDH-mutant cancers most likely contributes to cancer initiation via inhibition of TET2 and probably also other epigenetic pathways. However, in IDH-wild-type AML cells, 2HG inhibits cancer proliferation in general. In low grade glioma and subsets of IDH-mutant AML cases in which the presence of 2HG leads to a more benign outcome, we suggest that 2HG contributes to cancer initiation via inhibiting TET2, but suppresses cancer progression/proliferation via inhibiting FTO/MYC signaling.^69^

## FTO and R-2HG mainly target internal m^6^A rather than 5′ cap m^6^A_m_ in leukemia

FTO has also been reported to demethylate 5′ cap *N*^6^,2′-*O*-dimethyladenosine (m^6^A_m_).^71^ However, we found that internal m^6^A abundance is approximately 20–30 times of the near 5′ cap m^6^A_m_ abundance in human AML cells as detected by liquid chromatography-tandem mass spectrometry (LC-MS/MS) assays, and R-2HG treatment or *FTO* knockdown or overexpression in leukemia cells mainly affects internal m^6^A abundance^69^ (Su et al., 2018). In addition, we analyzed our m^6^A-seq data from human AML cells and found that over 95% of the m^6^A peaks affected by R-2HG treatment or *FTO* knockdown or overexpression are internal m^6^A, not 5′ cap m^6^A_m_^69^ (Su et al., unpublished). We also analyzed the fold changes of m^6^A_m_, A_m_, C_m_, G_m_, and U_m_-initiated mRNAs in leukemia cells, and found that m^6^A_m_-initiated mRNAs showed an even smaller fold change in expression than the other four groups of mRNAs upon R-2HG treatment or *FTO* overexpression. Even if increased m^6^A_m_ through FTO inhibition by 2HG plays a role, it should lead to increased transcript stability,^71^ which is opposite to what was observed here for *MYC* and *CEBPA* as well as the observed cancer inhibition effect of 2HG.^69^ Moreover, our luciferase reporter and mutagenesis assays and gene-specific m^6^A-qPCR assays demonstrate that FTO demethylates the internal m^6^A, not potential cap m^6^A_m_, on target mRNA transcripts such as *ASB2*, *RARA*, *MYC*, and *CEBPA*.^55,69^ Taken together, the common internal m^6^A modifications, rather than the rare 5′ cap m^6^A_m_, are the main substrates of FTO that are responsible for FTO-mediated gene regulation and oncogenic role at least in leukemia. Of course, it is possible that m^6^Am might also be an important substrate of FTO in some other types of cells in which m^6^Am abundance is high.

## ALKBH5 plays an oncogenic role as an m^6^A eraser in brain tumor and breast cancer

**Fig. 3:**
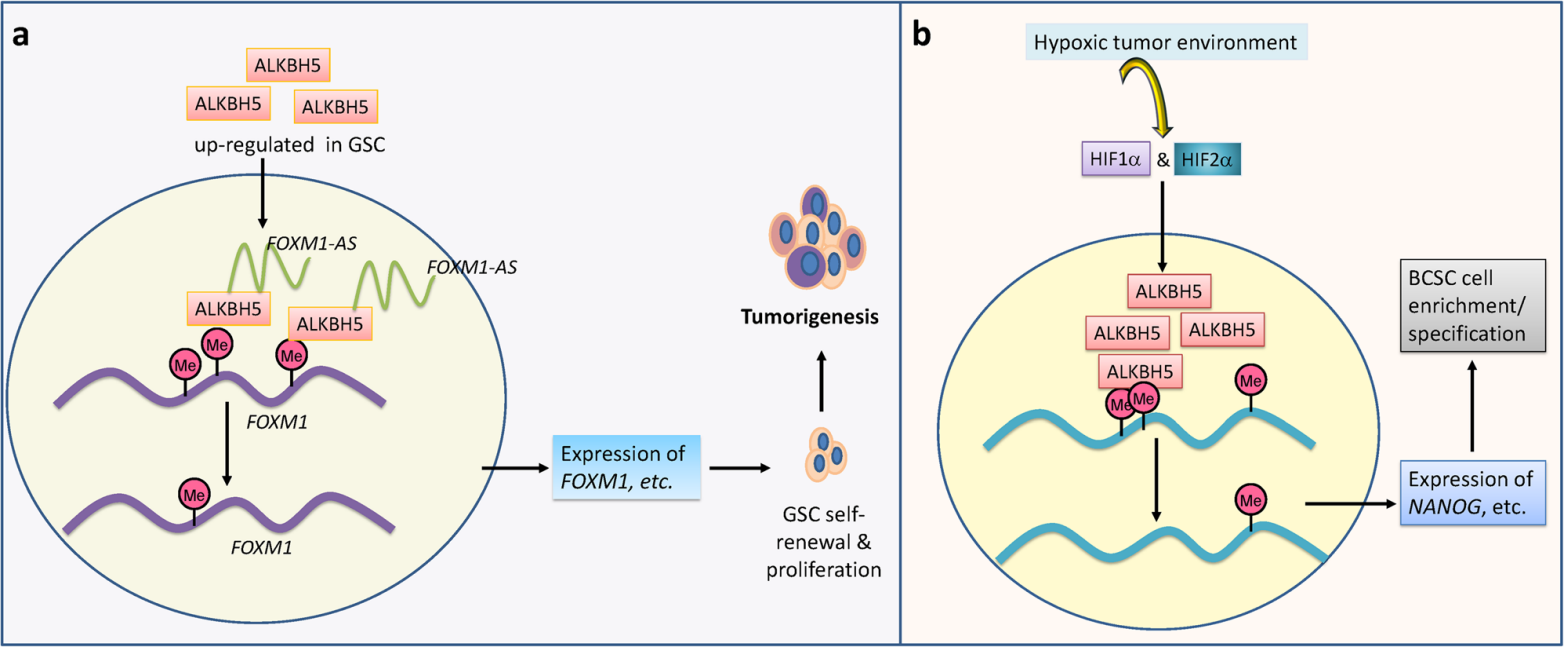
ALKBH5 plays an oncogenic role in brain tumor and breast cancer. **a** ALKBH5 enhances self-renewal and proliferation of GSCs and promotes tumorigenesis through regulating expression of FOXM1 with the aid of FOXM1-AS. **b** HIF-induced ALKBH5 expression mediates the upregulation of pluripotency factor expression and the enrichment/specification of BCSCs in the hypoxic environment

As the second m^6^A demethylase identified, ALKBH5 was reported by He and colleagues to affect mRNA export and RNA metabolism, and regulate spermatogenesis and apoptosis in mouse testes through targeting the p53 signaling pathway.^17^ Recently, it was reported that ALKBH5 functions as an oncoprotein in the pathogenesis of GBM and breast cancer, affecting the self-renewal and proliferation of relevant cancer stem cells.^72,73^ In brain tumors, *ALKBH5* expression is aberrantly upregulated in GSCs and its increased expression is associated with poor outcome in patients with GBM.^72^ Elevated expression of ALKBH5 enhances self-renewal and proliferation of GSCs, while depletion of *ALKBH5* expression significantly inhibits tumor development in nude mice intracranially implanted with GSCs.^72^ Mechanistically, ALKBH5 functions as an m^6^A demethylase, and enhances expression of its key target, *FOXM1*, by reducing m^6^A abundance on target mRNA transcripts (especially at the 3′ UTR); meanwhile, *FOXM1-AS*, a long non-coding RNA (lncRNA) that is located opposite to *FOXM1* on human chromosome 12 with partial overlap, facilitates the interaction between ALKBH5 and nascent transcripts of *FOXM1*. As a functionally important target of ALKBH5, *FOXM1* overexpression can reverse the effects of *ALKBH5* or *FOXM1-AS* knockdown and reinstate the tumor growth of GSCs.^72^ Collectively, this study reveals that ALKBH5 enhances self-renewal and proliferation of GSCs and promotes tumorigenesis through regulating expression of *FOXM1*, with the aid of *FOXM1-AS*^72^ (see Fig. 3a).

It was also reported that hypoxia-stimulated HIF1α and HIF2α promote the expression of *ALKBH5* in hypoxic breast cancer cells, and elevated expression of ALKBH5 promotes mRNA stability and expression of *NANOG*, a gene encoding a pluripotency factor, by catalyzing m^6^A demethylation.^73^ Ectopic expression of *ALKBH5*, under nonhypoxic conditions, significantly enhances *NANOG* expression and promotes enrichment of breast cancer stem cells (BCSCs), phenocopying the effects of hypoxia. Conversely, knockdown of *ALKBH5* impairs hypoxia-induced *NANOG* expression and BCSC enrichment, and also impairs tumor formation in vivo.^73^ Thus, HIF-induced ALKBH5 expression mediates the upregulation of pluripotency factor expression and the enrichment/specification of BCSCs in the hypoxic tumor microenvironment through negative modulation of RNA methylation (see Fig. 3b). The same group showed further that both ALKBH5 and ZNF217 participate in the hypoxia-induced *NANOG* and *KLF4* (another pluripotency factor gene) overexpression in breast cancer cells.^74^

## METTL14 and METTL3 regulate normal and malignant hematopoiesis as m^6^A writers

**Fig. 4:**
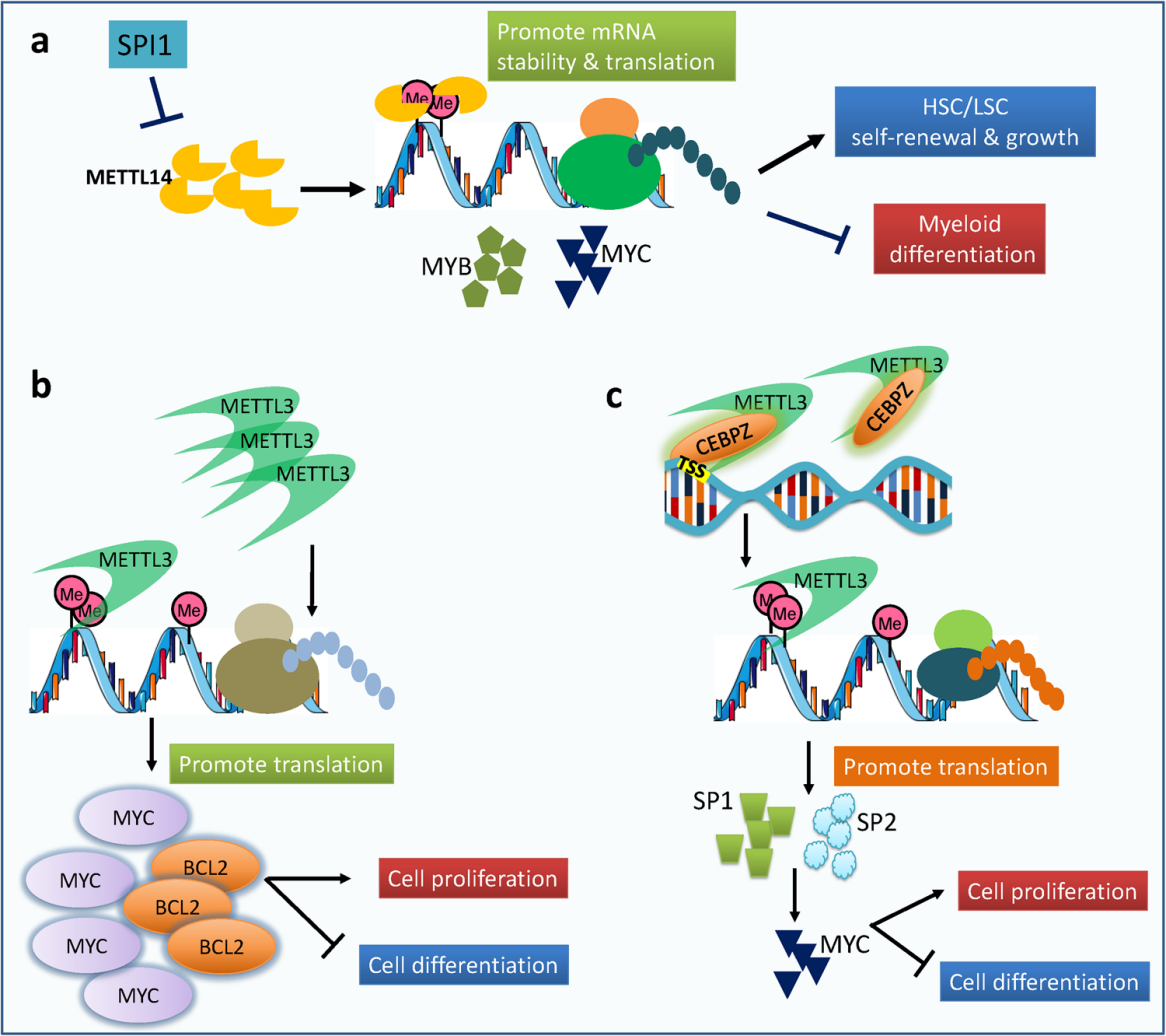
METTL14 and METTL3 play oncogenic roles in leukemia. **a** METTL14 plays an essential oncogenic role in AML development and maintenance through regulating expression of critical targets (e.g., *MYB* and *MYC*) via an m^6^A-dependent mechanism. **b** METTL3 promotes AML cell proliferation and inhibits myeloid differentiation likely through promoting translation of its potential mRNA targets (e.g., *MYC*, and *BCL2*). **c** METTL3 is recruited to TSSs of target genes by CEBPZ, and its potential direct targets are SP1 and SP2, which regulate expression of *MYC*

As two major components of m^6^A MTC, the functions of METTL14 and METTL3 in normal and malignant hematopoiesis have been reported recently. We found that METTL14 is highly expressed in normal HSPCs and is downregulated during myeloid differentiation, and depletion of *METTL14* expression further promotes terminal myeloid differentiation of normal HSPCs.^75^ METTL14 is also aberrantly overexpressed in human AMLs, especially those carrying t(11q23), t(15;17) and t(8;21), and forced expression of individual oncogenic fusion proteins resulting from such chromosomal translocations leads to upregulation of *METTL14* expression. Moreover, we have demonstrated that METTL14 is required for both initiation and maintenance of AML and self-renewal of leukemia stem/initiation cells (LSCs/LICs).^75^ Mechanistically, METTL14 exerts its oncogenic role through m^6^A-dependent post-transcriptional regulation of its critical mRNA targets such as *MYB* and *MYC*, which encode master transcriptional regulators that are essential for self-renewal of normal HSPCs and LSCs/LICs;^76–80^ expression of *METTL14* itself is negatively regulated by SPI1 (PU.1), a transcriptional master regulator of myelopoiesis.^81^ Notably, METTL14 promotes expression of *MYB* and *MYC* by increasing m^6^A abundance and enhancing stability of the target mRNA transcripts and likely also enhancing their translation.^75^ Collectively, our studies demonstrate that METTL14 plays an essential role in normal hematopoiesis and especially AML development and maintenance through blocking myeloid differentiation and promoting self-renewal of normal HSPCs and LSCs/LICs via an m^6^A-dependent mechanism involving the SPI1⊣METTL14-m^6^A-MYB/MYC signaling axis (see Fig. 4a).^75^ Our work also suggests that targeting METTL14, especially in combination with differentiation-inducing agents, may represent effective novel therapeutic strategies to treat AMLs with high levels of METTL14.^75^

As the main m^6^A methyltransferase, METTL3 has been shown recently to play a critical role in cell fate determination during the endothelial-to-hematopoietic transition (EHT) to specify the earliest HSPCs in vertebrate embryogenesis through an m^6^A-dependent mechanism.^36^ In zebrafish embryos, mettl3 is enriched in sorted endothelial cells and hemogenic endothelium, and loss-of-function of *mettl3* by morpholino treatment^14^ or genetic knockout caused a significant decrease of m^6^A and a block of the emergence of HSPCs; a similar phenotype was observed in mice when *Mettl3* was knocked down.^36^ Mechanistically, mettl3 deficiency causes continuous activation of Notch signaling, due to the suppression of YTHDF2-mediated mRNA decay of *notch1a* and *rhoca* in arterial endothelial cells, which in turn blocks EHT and thereby represses the generation of the earliest HSPCs.^36^

It was also reported recently that METTL3 plays an essential role in controlling myeloid differentiation of mammalian normal hematopoietic and leukemic cells.^82^ Forced expression of wild-type METTL3, but not a mutant METTL3 (with defect in catalytic activity), significantly promotes cell proliferation and inhibits cell differentiation of human cord blood-derived CD34 + HSPCs; the opposite is true when *METTL3* is knocked down by shRNAs. METTL3 is highly expressed in AML compared to normal HSPCs or other types of cancers. Knockdown of *METTL3* in human AML cell lines significantly induces cell differentiation and apoptosis and inhibits leukemia progression in mice xeno-transplanted with MOLM-13 AML cells. The biological function of METTL3 is likely attributed to the promotion of translation of its mRNA targets such as *MYC*, *BCL-2*, and *PTEN* in an m^6^A-dependent manner, although the exact molecular mechanism has not yet been defined (see Fig. 4b).^82^

A more recent study also demonstrated that METTL3 is critical for the maintenance of myeloid leukemia state.^83^ Interestingly, Barbieri et al.^83^ showed that METTL3 and METTL14 can both bind to chromatin, but mainly localize to the transcription start sites (TSSs) of distinct sets of coding genes that are featured with bimodal H3K4me3 peaks. The recruitment of METTL3 to TSSs of target genes is mediated by CEBPZ, a CCAAT-box binding factor. Promoter-bound METTL3 is required for m^6^A modification of associated transcripts, and METTL3 regulates translation, rather than transcription, of the relevant target transcripts.^83^ SP1 and SP2, which regulate expression of *MYC*,^84^ are two potential direct targets of METTL3 (see Fig. 4c).^83^

## The functions of METTL3 and METTL14 in GBM and liver cancer are controversial

In GBM, Cui et al.^60^ reported that consistent with the increased m^6^A levels during the differentiation of GSCs, overexpression of wild-type METTL3, but not a catalytically inactive mutant of METTL3, significantly promoted differentiation of GSCs and inhibited the self-renewal and proliferation of GSCs. Conversely, depletion of *METTL3* or *METTL14* expression significantly enhanced GSC growth and self-renewal in vitro and promoted tumor progression in vivo.^60^ A number of GSC-associated genes (e.g., *ADAM19*) are putative targets of m^6^A modifications in GSCs that are probably responsible for the phenotypes caused by manipulating the expression of individual m^6^A writer or eraser genes.^60^ However, the opposite role of METTL3 in GBM was reported by a different group.^85^ They showed that *METTL3* is highly expressed in GSCs and is downregulated during differentiation, associated with decreased levels of m^6^A during differentiation; silencing of *METTL3* expression in GBM significantly inhibited tumor growth in mice and prolonged mouse survival, which is consistent with the observation that elevated expression of *METTL3* was associated with poor survival in GBM patients; *METTL3* knockdown also sensitized GSCs to γ-irradiation.^85^ *SOX2* was identified as a functionally important target of METTL3, and METTL3-mediated m^6^A modification of *SOX2* mRNA transcripts makes them more stable. Overall, this study suggests that METTL3 plays a critical oncogenic role in GSC maintenance and radioresistance.^85^

In liver cancer, Ma et al.^86^ reported that METTL14 plays a tumor-suppressor role in hepatocellular carcinoma (HCC), in which METTL14 and m^6^A levels were decreased compared to normal tissue or paratumor controls, with largely unchanged levels of METTL3 and WTAP. In analysis of 130 in-house HCC patient samples, they found that decreased expression of *METTL14* was associated with poor prognosis in the patients; METTL14 knockdown enhanced HCC metastasis, and forced expression of METTL14 substantially suppressed HCC tumor invasion and metastasis, likely through m^6^A-dependent modulation of primary microRNA (e.g., mir-126) processing by interaction with DGCR8.^86^ In contrast, Chen et al.^87^ reported that METTL3 level was significantly higher and METTL14 level was slightly higher in HCC than in normal tissue, while WTAP level was unchanged; in analysis of TCGA HCC cohort dataset, they found that increased expression of *METTL3* was associated with poor prognosis in the patients. They further showed that overexpression of METTL3 significantly promoted growth of HCC both in vitro and in vivo, while depletion of METTL3 expression substantially inhibited tumorigenesis and lung metastasis of HCC in vivo, likely through negative regulation of *SOCS2* expression by an m^6^A- and YTHDF2-dependent mechanism.^87^ Similarly, they showed that METTL14 knockdown significantly suppressed HCC cell proliferation, migration and colony formation, and the opposite is true when METTL14 was overexpressed.^87^ Thus, they demonstrated that both METTL14 and METTL3 play oncogenic roles in HCC and are required for HCC growth and metastasis.^87^

## METTL3 plays an oncogenic role in lung cancer as a potential m^6^A reader

**Fig. 5:**
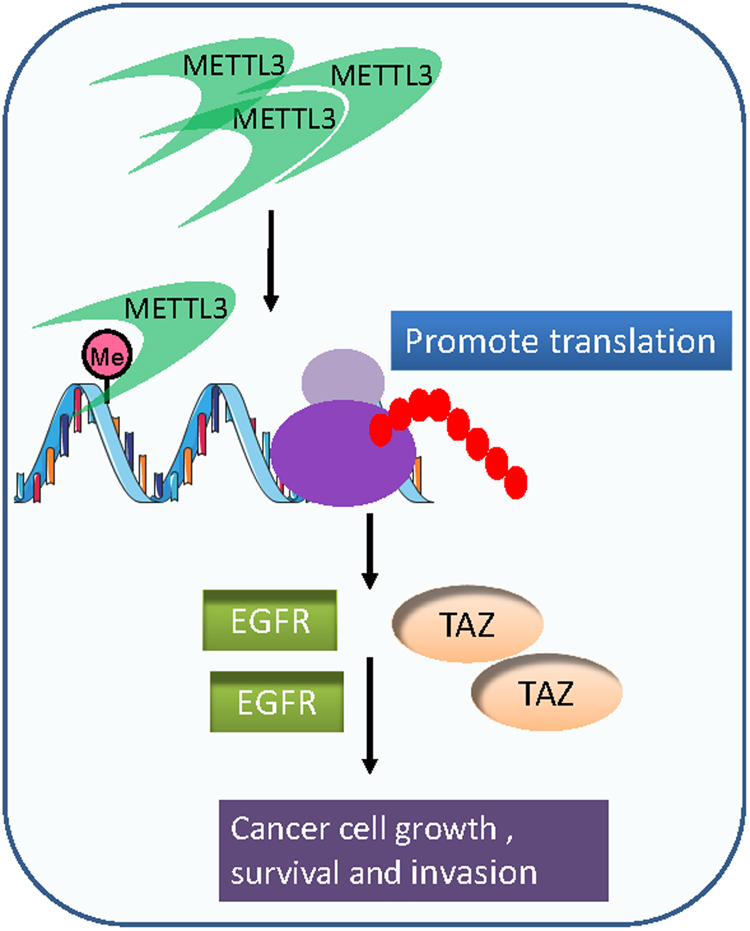
METTL3 plays an oncogenic role in lung cancer. METTL3 enhances the growth, survival, and invasion of lung cancer cells through promoting translation of target mRNA transcripts (e.g., *EGFR* and *TAZ*)

METTL3 was also reported to be upregulated in lung adenocarcinoma and play an oncogenic role in promoting the growth, survival and invasion of human lung cancer cells.^27^ Interestingly, this study suggests that METTL3 may function as an m^6^A reader in cytoplasm and promote translation of its target mRNA transcripts (e.g., *EGFR* and *TAZ*) by interaction with the translation initiation machinery^27^ (see Fig. 5). Nevertheless, METTL3′s catalytic activity might be still required for its function in promoting translation of m^6^A-containing target transcripts, because its targets need to be modified with m^6^A in nucleus first before their translation is enhanced in cytoplasm.

## IGF2BP proteins play oncogenic roles in cancers as m^6^A readers

Thus far, the most well documented m^6^A readers are the YTH domain-containing proteins including YTHDF1, YTHDF2, YTHDF3, YTDHDC1, and YTHDC2. Of them, YTHDF2, YTHDF3, and YTHDC2 promote decay of m^6^A-modified mRNAs.^18,20,23^ Interestingly, in contrast to what could be predicted by the mRNA decay mechanism mediated by YTHDF2, YTHDF3, or YTHDC2, our recent data showed that the vast majority portion of mRNA transcripts with a significant decrease in m^6^A abundance caused by overexpression of FTO tend to be downregulated in leukemia cells, likely due to decreased RNA stability along with reduced m^6^A abundance.^55^ In addition, through analysis of publically available datasets and our own experimental datasets, we found that a significant proportion of m^6^A-modified mRNA transcripts tend to be downregulated upon knockdown of m^6^A writers (*METTL3* and/or *MELLT14*). Thus, we presumed that there could be alternative m^6^A reader(s) that promote mRNA stabilization.

Indeed, through both m^6^A-oligo-pulldown/mass spectrometry assays and in silico m^6^A-binding protein prediction analysis, we have recently identified the insulin-like growth factor-2 (IGF2) mRNA-binding proteins 1, 2, and 3 (IGF2BP1/2/3) as a new family of m^6^A readers, which selectively recognize m^6^A-modified mRNAs with a consensus of GG(m^6^A)C^25^. We show that IGF2BPs promote the stability and storage of their target mRNAs (e.g., *MYC*, *FSCN1*, *TK1*, and *MARCKSL1*) in an m^6^A-dependent manner in normal and stress conditions, likely by recruiting mRNA stabilizers such as HuR and MATRIN3. Different from the previously identified m^6^A reader proteins that contain a YTH domain,^9,18,21,88^ IGF2BP proteins contain six canonical RNA-binding domains, including two RNA recognition motif domains on the N-terminus and four KH domains on the C-terminal regions.^89,90^ Our data indicate that the KH3-4 di-domains of IGF2BP proteins are the most critical domains for their binding to m^6^A-modified target mRNAs and for their biological functions. Remarkably, over 3000 mRNA transcripts were identified as targets of each individual IGF2BP proteins, with over 5000 mRNAs being targeted by at least one protein and more than 2000 being co-targeted by all three IGF2BP proteins, highlighting the broad impact of the IGF2BP proteins as m^6^A readers that globally regulate gene expression at the post-transcriptional level. Notably, the binding sites of IGF2BP proteins are highly enriched in the 3′ end of target mRNAs. In addition, our data suggest that IGF2BP proteins are likely also involved in translation initiation/elongation of target mRNAs.^25^

**Fig. 6:**
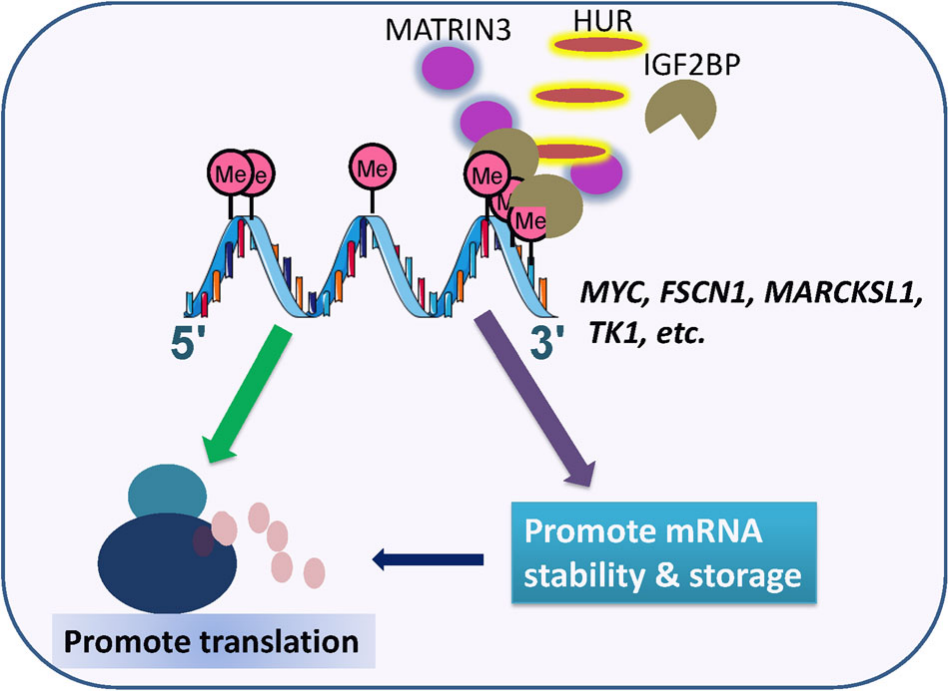
IGF2BP1/2/3 proteins play oncogenic roles in cancers. IGF2BP1/2/3 proteins promote proliferation, migration, and invasion of cancer cells through post-transcriptionally regulating the stability and translation of key target mRNAs (e.g., *MYC*)

We also showed that knockdown of individual *IGF2BP* genes significantly inhibited cell growth/proliferation, colony formation, and migration and invasion of human cervical cancer (Hela) and liver cancer (HepG2) cells. Such function of IGF2BP proteins relies on their role as m^6^A readers. *MYC* is a critical target of IGF2BPs in cancers, and its depletion mimics the phenotypes caused by *IGF2BP* depletion while its overexpression can rescue the effects of *IGF2BP* depletion.^25^ Collectively, IGF2BPs elicit oncogenic functions as m^6^A readers in promoting proliferation, migration, and invasion of cancer cells through post-transcriptionally regulating the stability and also translation of their key target mRNAs (e.g., *MYC*). Our work reveals a new facet of m^6^A reading and also suggests IGF2BPs as potential targets for anti-cancer therapy (see Fig. 6).

## Conclusions and perspectives

Despite still being in the infant stage, recent studies of m^6^A in cancers have revealed that m^6^A modification and the associated regulatory proteins play critical roles in a variety of cancers (see Table 1 for a summary). The m^6^A writers and erasers, relative to readers, have been better studied in cancers. Interestingly, a given m^6^A regulatory protein may play a similar role across different types of cancers. For example, FTO functions as an oncoprotein in both leukemia and GBM^55,60^ and ALKBH5 plays an oncogenic role in both breast cancer and GBM.^72,73^ Notably, while the oncogenic roles of METTL3 and METTL14 in AML have been confirmed by different groups,^75,82,83^ their reported functions in brain and liver cancers are controversial.^60,85–87^ The different roles of a given gene (e.g., METTL3 and METTL14) in the same cancer type (e.g., GBM and HCC) reported by different groups might be due to genetic/epigenetic heterogeneities of the cancer cell lines and primary tumor specimens used by different groups, and thus further systematical studies are warranted to clarify the discrepancies and better understand the factors that affect the functions of a given gene in different cellular contexts.

**Table 1:** The roles and mechanism of m^6^A regulators in cancer

**Regulator**	**Function in cancer**	**m** ^**6**^ **A-related role**	**Functional mechanism**	**Refs.**
FTO	Oncogenic role in AML: promoting leukemogenesis and drug resistance	m^6^A eraser	Targeting *ASB2*, *RARA*, *MYC*, and *CEBPA*, etc; FTO itself is a target of 2HG	55,69
	Oncogenic role in GBM: pharmaceutical inhibition of FTO suppresses GBM development	m^6^A eraser	N/A	60
ALKBH5	Oncogenic role in GBM: promoting tumorigenesis and self-renewal/proliferation of GSCs	m^6^A eraser	Targeting *FOXM1*, etc	72
	Oncogenic role in breast cancer: promoting tumorigenesis and proliferation of BCSCs	m^6^A eraser	Probably targeting NANOG, etc	73
METTL14	Oncogenic role in AML: promoting LSC/LIC self-renewal and leukemogenesis and inhibiting myeloid differentiation	m^6^A writer complex component	Targeting *MYB* and *MYC*, etc	75
	Tumor-suppressor role in GBM: inhibiting tumorigenesis and self-renewal/proliferation of GSCs	m^6^A writer complex component	Probably targeting *ADAM19*, etc	60
	Tumor-suppressor role in HCC: inhibiting tumor invasion and metastasis	m^6^A writer complex component	Inhibiting primary microRNA (e.g., mir-126) processing	86
	Oncogenic role in HCC: promoting HCC cell proliferation and migration	m^6^A writer complex component	Targeting *SOCS2*, etc	8 7
METTL3	Oncogenic role in AML: promoting leukemogenesis and inhibiting myeloid differentiation	m^6^A methyltransferase	Probably targeting *MYC*, *BCL2, PTEN, SP1*, and *SP2*, etc	82,83
	Tumor-suppressor role in GBM: inhibiting tumorigenesis and self-renewal/proliferation of GSCs	m^6^A methyltransferase	Probably targeting *ADAM1*9, etc	60
	Oncogenic role in GBM: promoting tumorigenesis, GSC maintenance, and radioresistance	m^6^A methyltransferase	Targeting *SOX2*, etc	85
	Oncogenic role in HCC: promoting HCC cell proliferation and migration	m^6^A methyltransferase	Targeting *SOCS2*, etc	87
	Oncogenic role in lung cancer: promoting growth, survival and invasion of lung cancer cells	m^6^A reader?	Probably targeting *EGFR* and *TAZ*, etc	27
IGF2BP1/2/3	Oncogenic roles in cervical and liver cancer: promoting growth, colony formation, migration and invasion of cervical and liver cancer cells	m^6^A readers	Targeting *MYC*, *FSCN1*, *TK1*, and *MARCKSL1*, etc	25

*m*^*6*^*A* *N*^6^ methyladeosine, *AML* acute myeloid leukemia, *GBM* glioblastoma, *HCC* hepatocellular carcinoma, *LSC/LIC* leukemia stem/initiating cell, *GSC(s)* glioblastoma stem(-like) cell(s), *N/A* data not available

**Fig. 7:**
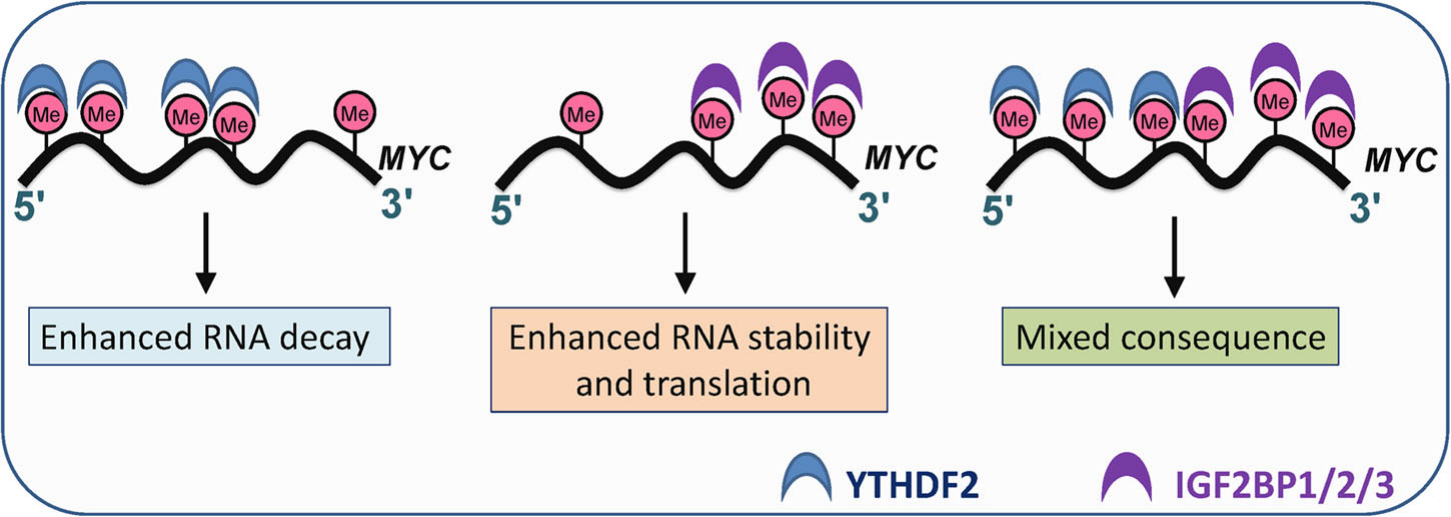
Model of YTHDF2- and IGF2BP1/2/3-mediated m^6^A-dependent post-transcriptional regulation of *MYC* expression. IGF2BP1/2/3 proteins preferentially bind to m^6^A sites in the 3′ end region of *MYC* and enhance RNA stability and promote RNA translation; in contrast, YTHDF2 protein preferentially binds to m^6^A sites in the 5′ end and middle regions of *MYC* and promotes RNA decay (based on Su et al., unpublished data)

One may expect that m^6^A writer and eraser proteins function oppositely in a given type of cancer. However, this is not always the case. For instance, while FTO plays an essential oncogenic role in AML as an m^6^A eraser,^55,69^ three components of the m^6^A MTC including METTL3,^82,83^ METTL14^75^, and WTAP^91^ also function as oncoproteins in AML. Consistent with this, it is well known that TET2 (a DNA demethylase) and DNMT3A (a DNA methyltransferase) both function as tumor suppressors in myeloid malignancies in which they both are frequently associated with loss-of-function mutations^92,93^; furthermore, they can work cooperatively in repressing lineage differentiation of hematopoietic stem cells.^94^ Therefore, it is not unusual that a writer and an eraser of the same epigenetic modification (e.g., m^6^A RNA modification or DNA methylation) may play similar functional roles in the same cancer cell context, probably through regulating distinct sets of target genes. Alternatively, they may also target the same set of genes and cause similar biological consequences through different mechanisms. Indeed, we found that *MYC* is a critical target of and positively regulated by both FTO and METTL14.^69,75^ FTO mainly modulates m^6^A abundance on the 5′-terminal and middle exons of *MYC* mRNA;^69^ in contrast, *METTL14* overexpression or depletion mainly affects m^6^A abundance in the 3′-region of *MYC*,^75^ likely due to the compensation effect of FTO on m^6^A modification of the other regions of *MYC* mRNA, because *FTO* expression is also positively regulated by METTL14 through an indirect mechanism (Su et al., unpublished data). There is a ~250- nucleotide *cis*-acting element termed as coding region instability determinant (CRD) in the 3′-region of *MYC*, which is required for regulating the stability of *MYC* mRNA.^95^ We showed that IGF2BP proteins preferentially recognize and bind to the m^6^A-modified CRD region of *MYC* mRNA, thereby stabilizing *MYC* mRNA and promoting translation;^25^ in contrast, YTHDF2 preferentially recognizes and binds to m^6^A-modified 5′-terminal and middle exons of *MYC* mRNA and thereby promotes mRNA decay^69^ (Su et al., unpublished data); this model is illustrated in Fig. 7. Moreover, while FTO preferentially recognizes and binds to m^6^A modifications on the 5′-terminal and middle exons of *MYC* mRNA, ALKBH5 preferentially recognizes and binds to m^6^A modifications on the 3′-region of *MYC* mRNA (Su et al., unpublished data). Interestingly, *ALKBH5* was reported previously to be frequently associated with DNA copy number loss in AML, especially in AML carrying p53 mutations, implying that it may play a tumor-suppressor role in AML.^96^ Overall, different m^6^A erasers and readers may preferentially bind to distinct regions of the same mRNA transcripts and lead to different fates of the target transcripts. For instance, while FTO promotes the stability of *MYC* mRNA through inhibition of YTHDF2-mediated RNA decay due to decreased m^6^A abundance on the 5′-terminal and middle exons of *MYC* mRNA,^69^ METTL14 also promotes the stability and translation of *MYC* mRNA through IGF2BPs-mediated RNA stability/translation enhancement due to increased m^6^A abundance on the 3′-region of *MYC* mRNA.^25,75^ Similarly, METTL3 was also shown to be able to promote translation of *MYC* mRNA^82^ and also probably indirectly regulate MYC transcription.^83^

A number of target genes of the aforementioned m^6^A regulators have been identified or implicated (see Table 1), and their expression is post-transcriptionally affected by the m^6^A regulators through m^6^A-dependent mechanisms, such as increased RNA decay or stability, and/or enhanced RNA translation. Many of such targets have been validated to be functionally important targets that upon appropriate manipulations can largely mimic or rescue the phenotype caused by the manipulation of a given m^6^A regulator. It is always very important to identify the most essential targets that are largely or even fully responsible for the effects of manipulation of a given m^6^A regulator. On the other hand, it would also be important to better understand the global effects of manipulation of individual m^6^A regulators, which may affect expression of hundreds or even thousands of downstream targets.

In addition, as the fates of m^6^A-modified RNA transcripts are ultimately determined by the types of m^6^A reader proteins that recognize and bind to the transcripts, it would be also important to identify the reader proteins that bind to and regulate expression of the functionally important targets. Actually, different readers may target distinct sets of transcripts, but in some cases different readers may preferentially bind to distinct regions of the same transcripts or even competitively bind to the same regions of the same transcripts. Therefore, in order to better understand m^6^A-mediated regulation of mRNA transcripts, it would be important to know which regions of the mRNA transcripts are m^6^A-modified and what type(s) of readers bind to the modified region(s).

The important roles of m^6^A regulatory proteins observed in various cancers suggest that they are potential therapeutic targets of cancer therapy. For example, given the essential role of FTO in leukemia and GBM,^55,60,69^ targeting FTO holds therapeutic potential to treat such cancers. Indeed, several FTO small-molecule inhibitors have been developed to inhibit the catalytic activity of FTO.^59,97–99^ MA^59^ has been shown to be able to inhibit GBM tumor progression in vivo.^60^ We showed that by inhibition of FTO catalytic activity and expression, 2HG can significantly suppress survival/proliferation of leukemic cells in vitro and substantially inhibit leukemia progression in vivo.^69^ Therefore, either FTO inhibitors or 2HG (or its analogs) can be applied to the clinic to treat IDH1/2 wild-type GBM and leukemia, especially those with *FTO* overexpression; in treating IDH-mutant cancers, combinational application of both IDH-mutant inhibitors and FTO inhibitors could lead to a more beneficial outcome than using IDH-mutant inhibitors alone, as suppression of R-2HG production by IDH-mutant inhibitors alone may cause rebounded expression/function of FTO and thus may lead to relapse.

With regard to METTL3, the situation is more complicated. METTL3 was reported to play an oncogenic role in both AML^82^ and lung cancer.^27^ Nonetheless, METTL3 may also have other functions independent of its catalytic activity in lung cancer, although such function was not reported in AML.^27,82,83^ Thus, development of inhibitors to target METTL3’s catalytic activity may not be sufficient to inhibit its overall functions.

In the future, development of more selective and potent inhibitors for FTO and other m^6^A regulatory proteins may lead to the development of effective novel therapeutic strategies to treat various cancers. In particular, the combinations of such inhibitor(s) with other therapeutic agents may represent more effective therapies to treat cancers that are resistant to currently available therapies. Indeed, we found that there is a synergistic effect between R-2HG and standard therapeutic agents such as ATRA, AZA, Decitabine, and Daunorubicin.^69^ Consistently, it was reported previously that leukemia patients with IDH mutations are more sensitive to treatment with AZA or Decitabine,^100^ ATRA,^101^ or standard chemotherapy (e.g., Daunorubicin),^102,103^ than those without. Similarly, our data^69^ and previous studies^104^ showed that glioma cells carrying IDH mutations are also more sensitive to Temozolomide, a common chemotherapy agent for brain tumor treatment. Therefore, it is important to test different combinations for different types of cancers to achieve the optimal therapeutic effects with minimal side effects in a manner of precision medicine.
